# Complete Chloroplast Genome Sequence of* Coptis chinensis* Franch. and Its Evolutionary History

**DOI:** 10.1155/2017/8201836

**Published:** 2017-06-18

**Authors:** Yang He, Hongtao Xiao, Cao Deng, Gang Fan, Shishang Qin, Cheng Peng

**Affiliations:** ^1^College of Medical Technology, Chengdu University of Traditional Chinese Medicine, Chengdu 611137, China; ^2^Department of Pharmacy, Sichuan Academy of Medical Sciences & Sichuan Provincial People's Hospital, School of Medicine, University of Electronic Science and Technology of China, Chengdu 610072, China; ^3^Department of Bioinformatics, DNA Stories Bioinformatics Center, Chengdu 610000, China

## Abstract

The* Coptis chinensis* Franch. is an important medicinal plant from the Ranunculales. We used next generation sequencing technology to determine the complete chloroplast genome of* C. chinensis*. This genome is 155,484 bp long with 38.17% GC content. Two 26,758 bp long inverted repeats separated the genome into a typical quadripartite structure. The* C. chinensis* chloroplast genome consists of 128 gene loci, including eight rRNA gene loci, 28 tRNA gene loci, and 92 protein-coding gene loci. Most of the SSRs in* C. chinensis* are poly-A/T. The numbers of mononucleotide SSRs in* C. chinensis* and other Ranunculaceae species are fewer than those in Berberidaceae species, while the number of dinucleotide SSRs is greater than that in the Berberidaceae.* C. chinensis* diverged from other Ranunculaceae species an estimated 81 million years ago (Mya). The divergence between Ranunculaceae and Berberidaceae was ~111 Mya, while the Ranunculales and Magnoliaceae shared a common ancestor during the Jurassic, ~153 Mya. Position 104 of the* C. chinensis ndhG* protein was identified as a positively selected site, indicating possible selection for the photosystem-chlororespiration system in* C. chinensis*. In summary, the complete sequencing and annotation of the* C. chinensis* chloroplast genome will facilitate future studies on this important medicinal species.

## 1. Introduction

Chinese goldthread,* Coptis chinensis* Franch., is an important medicinal plant in the Ranunculaceae.* C. chinensis* is native to China and has been used in traditional Chinese medicine for centuries [1, 2]. The major active compounds of* C. chinensis* are protoberberine alkaloids [1], such as berberine, palmatine, jatrorrhizine, coptisine, columbamine, and epiberberine. These compounds have antiviral, anti-inflammatory, and antimicrobial activity, and they dispel dampness, remove toxicosis, and aid detoxification [3–6]. Despite the prominent roles of* C. chinensis* in medicine, understanding of its biology and evolution is limited due to a lack of genomic resources.

Chloroplast genomes in angiosperms are mostly circular DNA molecules ranging from 115 to 165 kb in length [7]. They exhibit a conserved quadripartite structure consisting of one large single copy (LSC) region, one small single copy (SSC) region, and two copies of inverted repeats (IR). Due to their low levels of recombination and substitution rates compared to nuclear genomes, plant chloroplast genomes are valuable sources of genetic markers for phylogenetic analyses. Over 21 complete genomes of species within the Ranunculales have been sequenced and deposited in the NCBI database (as of August 2016), and these data can be used to study chloroplast genome evolution in the Ranunculales.

The improvement of NGS technologies allows the sequencing of entire chloroplast genomes cheaper [8] and has resulted in the extensive use of chloroplast genomes for molecular marker and molecular phylogenetic studies. In our study, we assembled the complete* C. chinensis* chloroplast genome sequenced using the sequencing data generated by the Illumina HiSeq platform. Genome annotation reported both the conserved and variable information of the* C. chinensis* genome compared to other Ranunculales species. The phylogeny and molecular dating analyses also deepen our understanding of the evolutionary history of the Ranunculales order.

## 2. Materials and Methods

### 2.1. Plant Material and Library Preparation

*C. chinensis* was collected from Shizhu, Chongqing City, China. DNA extraction and library preparation used methods described by He et al. [8]. Fresh leaves were used to extract total chloroplast DNA with the Tiagen Plant Genomic DNA Kit (Beijing, China). 300*-*bp DNA fragments were obtained by breaking extracted genomic DNA using a Covaris M220 Focused*-*Ultrasonicator (Covaris, Woburn, MA, USA). NEBNext® Ultra™ DNA Library Prep Kit Illumina (New England, Biolabs, Ipswich, MA, USA) was used to construct a sequencing library according to the manual from the manufacturer.

### 2.2. DNA Sequencing, Data Preprocessing, and Genome Assembly

Cluster generation was performed using TruSeq PE Cluster Kit (Illumina, San Diego, CA, USA), and 2 × 100 bp reads were generated on an Illumina HiSeq 2500. FASTX-Toolkit (2016a) was used to remove the adaptor-contaminated reads, low-quality bases (quality scores <20 or ambiguous nucleotide) dominated reads, and short reads (<20 bp). The remaining reads were called “clean reads.” Velvet v1.2.07 [9] was used for the de novo assembly of these clean reads, with the parameters described by He et al. [10]. To determine the contig orders and orientations, the 43 Velvet contigs were then aligned to the* M. saniculifolia* chloroplast genome [11] (NCBI RefSeq accession NC_012615.1, a species in the Ranunculaceae). Then, five pairs of primers linking adjacent contigs were designed and used to perform PCR amplification of the unassembled regions, and the PCR products were sequenced with Sanger method. Finally, using the Lasergene SeqMan program from DNASTAR (Madison, WI, USA), the Sanger reads, together with the Velvet contigs, were further assembled into high-quality complete chloroplast genome (NCBI GenBank accession: KY120323).

### 2.3. Genome Annotation

The* C. chinensis* genomes were annotated with the DOGMA (Dual Organellar GenoMe Annotator) [12], followed by being manually reviewed to remove duplicated annotations and checking for start and stop codons. The predicted genes were also BLASTed [13] to the nonredundant protein sequences database from the NCBI, the KEGG [14], and the COG [15] database. The graphical illustration of the circular plastome was drawn using the GenomeVx [16]. To compare the function of chloroplast proteins from Ranunculales species, we annotated these proteins from Supplementary Table S3 against COG [15] database with the method same as* C. chinensis* (see Supplementary Material available online at https://doi.org/10.1155/2017/8201836).

### 2.4. SSR Identification

MISA (MIcroSAtellite identification tool, 2016b) was used to identify simple sequence repeats (SSRs) in the* C. chinensis *chloroplast genome together with 23 other chloroplast genomes. The settings included the following: more than 10 repeats for mononucleotide SSRs, six repeats for dinucleotide SSRs, five repeats for trinucleotide SSRs, five repeats for tetranucleotide SSRs, five repeats for pentanucleotide SSRs, and five repeats for hexanucleotide SSRs. Compound SSRs were defined as two SSRs with <100 nt interspace nucleotides.

### 2.5. Phylogenetic Tree Reconstruction and Divergence Time Estimation

The chloroplast genome annotation data from the species listed in Supplementary Table S3 were downloaded from NCBI. Then, genes existing in all 24 chloroplast genomes were exacted, and a total of 42 genes remained. Using the MUSCLE (version: v3.8.31, parameters: default) [17], the protein sequences from each gene were aligned. The CDS alignments were obtained by translating the corresponding protein alignments using PAL2NAL [18] and were further concatenated into a supermatrix. Using the CDS alignments dataset, the phylogenetic tree was reconstructed by the RAxML [19] with the GTR + Ι + Γ substitution model, and the divergence times were estimated by the MCMCTree program from the PAML4.7 package [20] following the methods described by He et al. [8]. 125 and 193 Mya were set as the lower and upper boundaries for the splitting of Magnoliaceae–Ranunculales clade [21].

### 2.6. Identification of Positively Selected Genes (PSGs)

The CDS alignments of 42 genes were used for the identification of positively selected genes. The *ω* (Ka/Ks) ratios of filtered reliable codons in 42 genes were calculated using the branch-site model of CODEML in PAML4.7a [20], setting* C. chinensis *as the foreground branch and the others as background branches. The null hypothesis was that *ω* of each site was either equal to 1 or less than 1, while the alternative hypothesis allows *ω* of particular sites on the foreground branch to be larger than 1. Then, likelihood ratio test (LRT) analyses were performed, and the *p* values were used to guide against violations of model assumptions. The branch was considered to have undergone positive selection if they showed a statistically significant LRT and positively selected sites on the branch were identified in the BEB analysis.

## 3. Results and Discussions

### 3.1. Genome Sequencing and Assembly

We generated 2.13 GB pair end reads (2 × 100 bp) using the Illumina HiSeq 2500 platform (Illumina, San Diego, CA, USA). Clean reads were obtained by removing adaptors and low-quality read pairs. In total, we got 10,624,225 clean read pairs, and these clean reads were assembled into 43 contigs with N50 length of 47,033 bp using Velvet assembler (Table 1). To determine the orders and orientations, these Velvet assembled contigs were aligned to the* Megaleranthis saniculifolia* chloroplast genome [11] (Supplementary Table S1), and then gaps between two adjacent contigs were closed by Sanger reads (Supplementary Figure S1; primers are listed in Supplementary Table S2). The final complete* C. chinensis* chloroplast genome is comprised of 155,484 bp with guanine-cytosine content of 38.17% and falls within the range of the typical angiosperm chloroplast genome. By comparing it with the* M. saniculifolia *chloroplast genome, we confirmed the synteny and the absence of reversions or disorders in the genome.

### 3.2. Genome Annotations

As the general quadripartite structure found in plant chloroplast genomes, the* C. chinensis* chloroplast genome has two inverted repeated regions (IRa and IRb) of 26,758 bp in length, which split the circular genome into small single copy (SSC) and large single copy (LSC) region with 17,383 and 84,585 bp lengths, respectively. We found that the guanine-cytosine content in LSC and SSC regions (36.4% and 32.1%, respectively) is less than that in IR regions (43%). The relatively higher GC content in IR regions may be attributable to the transfer-RNA genes and ribosomal-RNA genes, which is consistent with the results from* Pogostemon cablin *[10].

**Figure 1:**
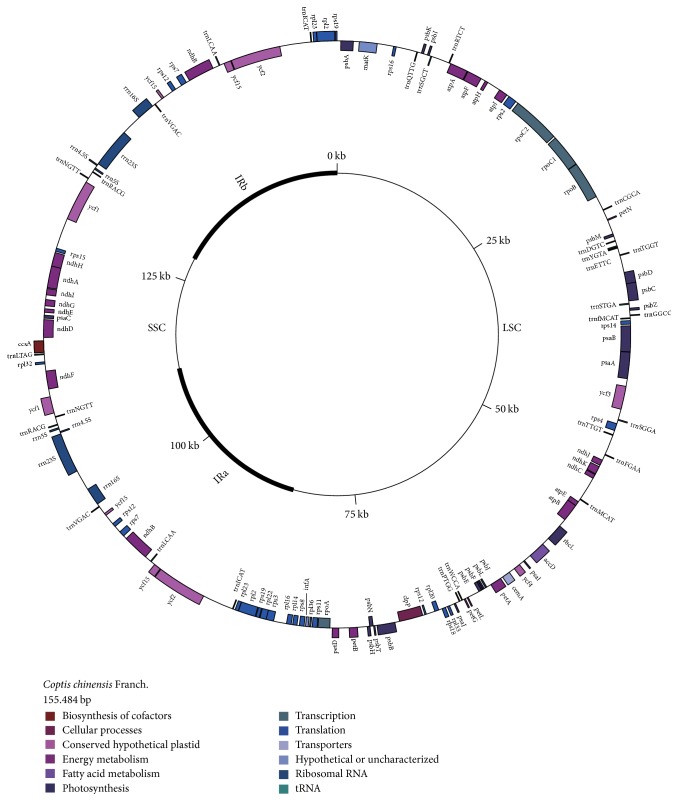
Genome schema of the* C. chinensis* chloroplast genome. Genes on the outer side the circle transcribe clockwise, while genes on the inner side transcribe counterclockwise. Genes from different functional groups are colored with different color.

The chloroplast genome of* C. chinensis* was predicted to consist of 128 gene loci, including 8 rRNA gene loci, 28 tRNA gene loci, and 92 protein-coding gene loci (Figure 1, Table 2). These gene loci contained 107 unique genes, including 80 protein-coding genes, 23 transfer-RNA genes, and 4 ribosomal-RNA genes. Each IR region contained five tRNA genes (including* trnI-CAT, trnL-CAA, trnV-GAC, trnR-ACG*, and* trnN-GTT*), nine protein-coding genes (ten loci), and all 4 rRNA genes. Extensions of the IR into the genes* rps19* and* ycf1* were identified (Figure 1) resulting in its pseudogenization due to incomplete duplication. There were 92 protein-coding gene loci, of which nine are duplicated (Table 2). The* ycf15 *gene has four copies in the* C. chinensis* chloroplast genome, and each IR region has two copies. The* rps12* gene has three copies, and IRa, IRb, and LSC region each have one copy (Table 2).

We mapped the proteins to the NR, Clusters of Orthologous Groups (COG) [15], and Kyoto Encyclopedia of Genes and Genomes (KEGG) [14] database. A total of 76 proteins were aligned to homologous orthologs in the KEGG database; only 56 proteins could be assigned to COG orthologs (Supplementary Tables S5-S6). Homologs of all 92 proteins except for two proteins from gene* ycf15* were identified in the NR database (Supplementary Table S4) showing high-quality annotation. Most of the proteins are involved in photosynthesis, energy metabolism, and ribosome-related functions, as indicated by annotations from the NR database and KEGG database. Consistent with other species from the same order, the COG classification of these proteins also mainly grouped into two groups: Category J (translation, ribosomal structure, and biogenesis) and Category C (energy production and conversion), which are in Supplementary Tables S7-S8.

### 3.3. Identification of Simple Sequence Repeats (SSRs)

We identified perfect SSRs in the* C. chinensis* chloroplast genomes, as well as the chloroplast genomes of several other species in the Ranunculales. We found that both the numbers and types of chloroplast SSRs are variable in different species (Table 3 and Supplementary Tables S10-S11). The most abundant SSRs in all the species were mononucleotide type, with numbers varying from 16 to 71. Moreover, most mononucleotide types are comprised of polyadenine and polythymine, which is consistent with the results of other studies [10]. In addition, mononucleotide SSRs in* C. chinensis* and other species in Ranunculaceae family were fewer than those in species from Berberidaceae family, while dinucleotide type was relatively more common.

### 3.4. Phylogenetic Tree Construction and Divergence Time Estimation

**Figure 2:**
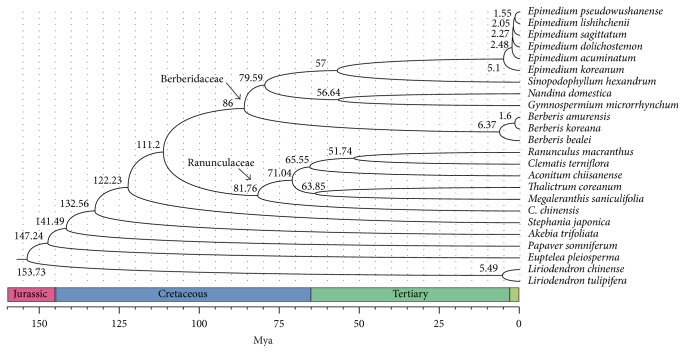
Phylogenetic tree and estimated divergence time based on chloroplast genomes from Ranunculales and Magnoliaceae.

To determine the evolutionary history of* C. chinensis* within the Ranunculales, we used 42 genes existing in all 24 chloroplast genomes, including 21 sequenced chloroplast genomes from species in the Ranunculales and two species from the Magnoliaceae as an outgroup. Phylogeny analysis shows that six Ranunculaceae species and 12 Berberidaceae plants comprise two unique clades, whereas the other four species are relatively divergent and ancestral in Ranunculales (Figure 2). Estimation of divergence times of these plants was performed using the MCMCTree program in the PAML4.7a package [20] (Figure 2), and all the times estimated matched well with the data deposited in TIMETREE, a public knowledge-base of divergence times among organisms, thereby confirming that the molecular clock dating strategy was reliable.* C. chinensis* is relatively ancestral in the Ranunculaceae and diverged from other Ranunculaceae plants about 81 million years ago (Mya). The divergence between Ranunculaceae and Berberidaceae was about 111 Mya, whereas Ranunculales and Magnoliaceae shared a common ancestor prior to divergence during the Jurassic period, around 153 Mya.

### 3.5. Selection in the Goldthread Chloroplast Genome

**Figure 3:**
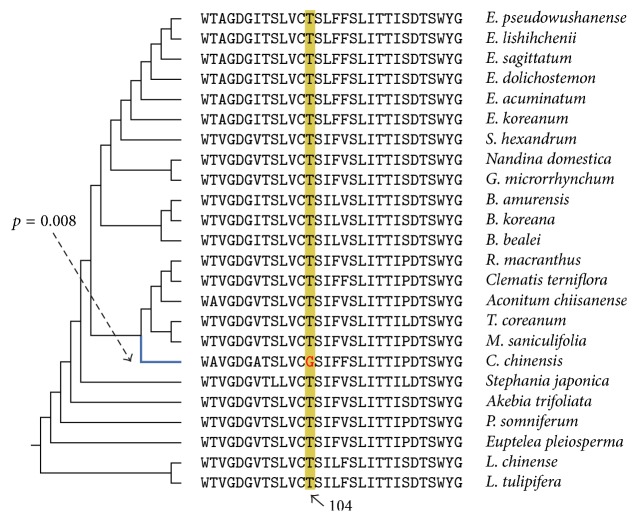
Multisequence alignments and positive selection of the* ndhG* gene. Blue branch is the foreground branch used in the branch-site test implemented, while yellow background color indicates the positively selected site (position 104 based on the* C. chinensis ndhG* protein sequence).

The extent to which the genes in the* C. chinensis* chloroplast genome have experienced selection is unknown. Therefore,* C. chinensis* genes and another 23 chloroplast genomes (Supplementary Table S3) were extracted and used to identify positively selected genes. The *ω* ratio (dN/dS, namely, nonsynonymous substitution rate/synonymous substitution rate) is used to measure the natural selection acting on a gene. To detect potential positive selection affecting selected sites along* C. chinensis* lineages, the branch-site model implemented in PAML [20] was applied (Figure 3). The results suggest that the* ndhG* (NADH dehydrogenase subunit 6) evolved under positive selection in the* C. chinensis* lineage (Supplementary Table S3). The test statistic (2ΔL) of* ndhG* gene was 5.79, and the *p* value was 0.008. BEB analysis revealed the position 104 of this protein as positively selected in* C. chinensis*, with posterior probabilities of 0.994. The* ndhG* is one of the 11 NADH dehydrogenase genes, and the* ndhG* subunit is associated with nuclear-encoded subunits to form the NADH dehydrogenase-like complex in angiosperm chloroplasts. This protein complex associates with photosystem I and then forms a supercomplex, which mediates cyclic electron transport [22], produces ATP to balance the ATP/NADPH ratio, and facilitates chlororespiration [23]. Therefore, the selection values identified in* C. chinensis* indicate positive selection for elements of the photosystem-chlororespiration system.

## Supplementary Material

Table S1. Mapping the contigs from goldthread chloroplast genome to the chloroplast genome of Megaleranthis saniculifolia. Table S2. The primers used for PCR during gapping closing. Table S3. Species used in this project. Table S4. Best hits with nr database of proteins in goldthread chloroplast genome. Table S5. Best hits with KEGG database of proteins in goldthread chloroplast genome. Table S6. Best hits with COG databases of proteins in goldthread chloroplast genome. Table S7. Best hits with COG database of chloroplast proteins from golthread and the other species. Table S8. The COG (Clusters of Orthologous Groups) classification and distribution of genes in different species. Table S9. LRT analysis of 42 genes in all 24 chloroplast genomes. Table S10. SSRs detected in goldthread and other species. Table S11. Detailed statistics of chloroplast SSRs detected in 24 species. Figure S1. PCR products on agarose gel electrophoresis. Each lane represents the PCR product of gap area (Table S2), except that the “1KbM” is the marker lane.

## Figures and Tables

**Table 1:** Genome sequencing and assembly of the *C. chinensis* chloroplast genome.

Sequencing	Raw data	2.13 G
	Raw reads (pair)	10,640,000
	Read length (bp)	2*∗*100
	Clean data	2.12 G
	Clean reads (pair)	10,624,225

Assembly	Total size	168,210 bp
	Contig num	43
	Average length	3,911 bp
	GC contents	38.77%
	N50 length	47,033 bp
	Min contig length	511 bp
	Max contig length	64,014 bp

Gap closing	Total size	155,484 bp
	Scaffold number	1
	GC contents	38.17%

**Table 2:** List of genes in the *C. chinensis* chloroplast genome. Numbers in the parentheses indicate the copy number in the genome.

Groups	Name of genes
Biosynthesis of cofactors	*ccsA*
Cellular processes	*clpP*
Conserved hypothetical plastid	*ycf1(2), ycf15(4), ycf2(2), ycf3, ycf4*
Energy metabolism	*atpA, atpB, atpE, atpF, atpH, atpI, ndhA, ndhB(2), ndhC, ndhD, ndhE, ndhF, ndhG, ndhH, ndhI, ndhJ, ndhK, petA, petB, petD, petG, petL, petN*
Fatty acid metabolism	*accD*
Hypothetical or uncharacterized	*infA, matK*
Photosynthesis	*psaA, psaB, psaC, psaI, psaJ, psbA, psbB, psbC, psbD, psbE, psbF, psbH, psbI, psbJ, psbK, psbL, psbM, psbN, psbT, psbZ, * *rbcL*
Ribosomal RNA	*rrn16S(2), rrn23S(2), rrn4.5S(2), rrn5S(2)*
Transcription	*rpoA, rpoB, rpoC1, rpoC2*
Translation	*rpl14, rpl16, rpl2(2), rpl20, rpl22, rpl23(2), rpl32, rpl33, rpl36, rps11, rps12(3), rps14, rps15, rps16, rps18, rps19(2), rps2, rps3, rps4, rps7(2), rps8*
Transporters	*cemA*
tRNA	*trnC-GCA, trnD-GTC, trnE-TTC, trnF-GAA, trnG-GCC, trnI-CAT(2), trnL-CAA(2), trnL-TAG, trnM-CAT, trnN-GTT(2), trnP-TGG, trnQ-TTG, * *trnR-ACG(2), trnR-TCT, trnS-GCT, trnS-GGA, trnS-TGA, trnT-GGT, * *trnT-TGT, trnV-GAC(2), trnW-CCA, trnY-GTA, trnfM-CAT*

**Table 3:** Statistics of chloroplast SSRs detected in 24 species. p1, mononucleotide SSRs; p2, dinucleotide SSRs; p3, trinucleotide SSRs; c, compound SSRs; A, adenine; G, guanine; T, thymine; C, cytosine.

Species	Total	p1					p2			p3	c
		All	(A)n	(C)n	(G)n	(T)n	All	(AT)n	(TA)n		
*E. pseudowushanense*	68	61	26	1	1	33	2	1	1	0	5
*E. lishihchenii*	73	67	26	1	1	39	2	1	1	0	4
*E. sagittatum*	63	55	22	1	1	31	2	1	1	0	6
*E. dolichostemon*	65	59	25	1	1	32	2	1	1	0	4
*E. acuminatum*	69	62	28	1	1	32	2	1	1	0	5
*E. koreanum*	68	60	26	1	1	32	2	2	0	0	6
*S. hexandrum*	33	27	10	0	1	16	2	2	0	0	4
*Nandina domestica*	52	48	19	0	0	29	1	0	1	0	3
*G. microrrhynchum*	71	59	26	1	0	32	1	0	1	1	10
*B. amurensis*	72	62	29	0	0	33	2	1	0	2	6
*B. koreana*	73	66	30	0	0	36	2	1	0	2	3
*B. bealei*	75	71	39	0	0	32	1	1	0	2	1
*R. macranthus*	32	28	13	0	0	15	3	3	0	1	0
*Clematis terniflora*	50	39	18	0	1	20	4	0	4	1	6
*Aconitum chiisanense*	34	21	9	2	0	10	10	4	6	0	3
*T. coreanum*	50	44	19	2	0	23	4	0	4	1	1
*M. saniculifolia*	38	36	10	0	0	26	2	2	0	0	0
*Coptis chinensis*	47	38	16	0	0	22	5	3	2	0	4
*Stephania japonica*	53	42	17	2	0	23	8	3	5	0	3
*Akebia trifoliata*	40	38	20	2	1	15	0	0	0	0	1
*P. somniferum*	16	16	8	0	0	8	0	0	0	0	0
*Euptelea pleiosperma*	65	57	27	0	0	30	0	0	0	0	8
*L. chinense*	49	41	13	2	0	26	3	2	1	1	4
*L. tulipifera*	56	47	18	2	0	27	3	2	1	1	5
