# Supplementary material for: Complete Chloroplast Genome Sequence of Coptis chinensis Franch. and Its Evolutionary History
Source: Biomed Res Int. 2017 Jun 18;2017:8201836. doi: 10.1155/2017/8201836 (PMC5494076; doi:10.1155/2017/8201836)
Supplement: Supplementary file 1 — Table S1. Mapping the contigs from goldthread chloroplast genome to the chloroplast genome of Megaleranthis saniculifolia. Table S2. The primers used for PCR during gapping closing. Table S3. Species used in this project. Table S4. Best hits with nr database of proteins in goldthread chloroplast genome. Table S5. Best hits with KEGG database of proteins in goldthread chloroplast genome. Table S6. Best hits with COG databases of proteins in goldthread chloroplast genome. Table S7. Best hits with COG database of chloroplast proteins from golthread and the other species. Table S8. The COG (Clusters of Orthologous Groups) classification and distribution of genes in different species. Table S9. LRT analysis of 42 genes in all 24 chloroplast genomes. Table S10. SSRs detected in goldthread and other species. Table S11. Detailed statistics of chloroplast SSRs detected in 24 species. Figure S1. PCR products on agarose gel electrophoresis. Each lane represents the PCR product of gap area (Table S2), except that the “1KbM” is the marker lane. [file 8201836.f1.zip › Supplementary Figures.pdf]

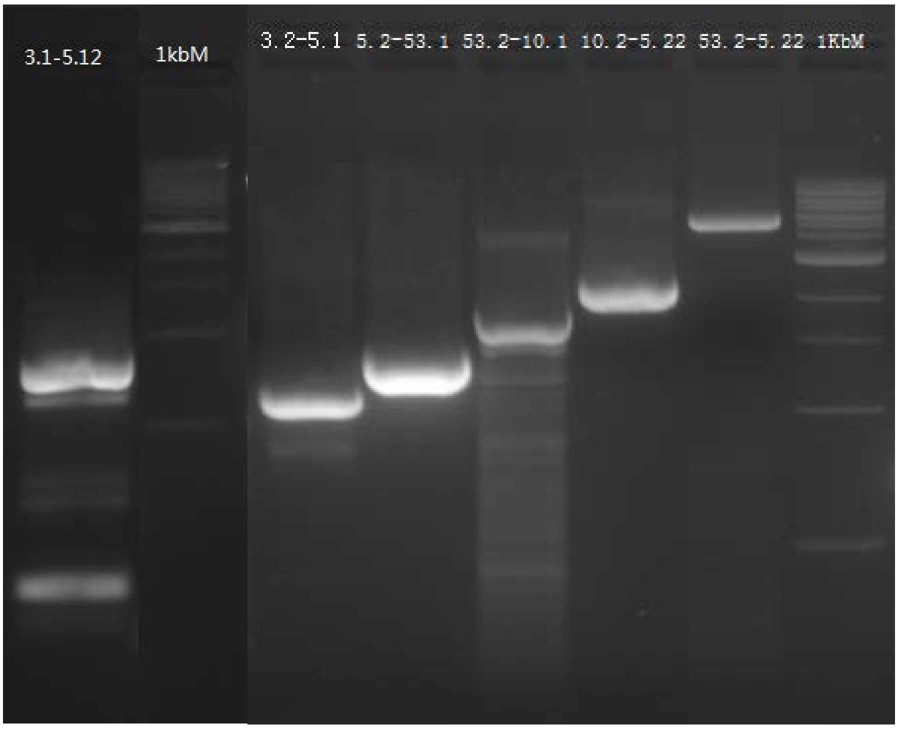

Figure S1. PCR products on agarose gel electrophoresis. Each lane represents the PCR product of gap area (Table S2), except that the “1KbM” is the marker lane.
